# Addressing Methodological Challenges in Follow-Up RCTs During the COVID-19 Pandemic: The Impact of the Good Behavior Game and MyTeachingPartner™ on Teacher Burnout and Self-Efficacy

**DOI:** 10.1007/s11121-024-01757-9

**Published:** 2025-01-02

**Authors:** Alexa C. Budavari, Heather L. McDaniel, Antonio A. Morgan-López, Rashelle J. Musci, Jason T. Downer, Nicholas S. Ialongo, Catherine P. Bradshaw

**Affiliations:** 1 University of Virginia School of Education and Human Development, Charlottesville, VA USA; 2 Research Triangle Institute International, Center for Behavioral Health and Well-Being, Raleigh, NC USA; 3 Johns Hopkins Bloomberg School of Public Health, Baltimore, MD USA; 4 Department of Psychology, University of South Carolina, Columbia, SC USA

## Abstract

Retention of early career teachers is a critical issue in education, with burnout and self-efficacy serving as important precursors to teachers leaving the field. An integration of the PAX Good Behavior Game (GBG; Barrish et al., 1969) and MyTeachingPartner (MTP; Allen et al., 2015) was tested in a randomized controlled trial (RCT) to investigate whether the combined programs would improve long-term outcomes for early career teachers. However, due to the COVID-19 pandemic, there was a higher proportion of missing data and attrition in follow-up data collection than otherwise would have been expected. The current paper focused specifically on intervention impacts on teacher-reported burnout and self-efficacy through the COVID-19 pandemic and explored various approaches for addressing missing data as an illustrative example for other researchers who may similarly have faced missing data challenges due to the pandemic. Participants included in the original trial were *N* = 188 early career teachers (grades K-3) who were randomly assigned to either the intervention (i.e., GBG + MTP; *n* = 94 teachers) or control condition (*n* = 94) and provided baseline data. Specifically, teachers reported on their burnout and self-efficacy at pre-intervention (Fall), post-intervention (Spring), 1-year post intervention (in Fall and Spring), COVID Year 1 (Spring 2021), and COVID Year 2 (Spring 2022). We conducted a series of outcomes analyses under varying missing data assumptions (i.e., MCAR, MAR, NMAR). There were mixed findings (i.e., both null and beneficial) regarding GBG + MTP impacts on burnout, which varied across missing data assumptions; however, there were no GBG + MTP impacts on self-efficacy. This study may also provide insight for other researchers encountering similar challenges when analyzing follow-up data collected during the COVID-19 pandemic, as we highlight pros and cons of several different approaches for modeling missing data related to attrition due to the COVID-19 pandemic and related school closures.

**Supplementary Information:**

The online version contains supplementary material available at 10.1007/s11121-024-01757-9.

Teacher retention is a critical problem in education, especially among early career teachers, with up to half of all teachers leaving the profession within the first five years (Chambers et al., 2019; Papay et al., 2017). One identified precursor to teachers leaving the field is poor occupational health, with early career teachers at increased risk for stress, burnout, and low self-efficacy, which has subsequent negative impacts on student academic and behavioral outcomes (Madigan & Kim, 2021; Skaalvik & Skaalvik, 2011, 2017). Given the association between student disruptive behavior, poor teacher occupational health, and high teacher turnover, the combined effects of two evidence-based programs focused on classroom management, the PAX Good Behavior Game (GBG; Barrish et al., 1969) and MyTeachingPartner™ (MTP; Allen et al., 2015) were tested in a randomized controlled trial (RCT) to investigate whether these programs can help improve long-term outcomes for early career teachers (e.g., retention, occupational health) and students (e.g., disruptive behavior). Initial findings from the study have shown promise of the preventive intervention among higher-risk teachers in classrooms with higher baseline levels of challenging student behaviors, with positive impacts on classroom management and teacher-student interactions, as well as teacher occupational health using pre-COVID follow-up observations (Downer et al., 2024; Tolan et al., 2020). The current study aimed to build on these initial findings by investigating the impact of the preventive intervention on teacher self-efficacy and burnout during the COVID pandemic. However, the pandemic resulted in significant sample attrition and other study-related challenges (e.g., inability to conduct classroom observations), that led us to focus on a narrower set of teacher-related outcomes and explore a number of different methodological approaches for managing the missing data. Further, the context surrounding the COVID-19 pandemic may have resulted in changes in the way teachers report on their occupational health; as such, we also accounted for potential differential item function in the outcome measures over time. The results of this analysis and contrasting different approaches for handling missingness in the follow-up study will inform the current study, as well as other RCTs impacted by COVID or experiencing similar missing data challenges.

## Teacher Self-Efficacy and Burnout

Teacher self-efficacy is a teacher’s own assessment of their ability to successfully complete teaching-related tasks, which is comprised of (1) their self-perception of teaching competence, and (2) their context-specific beliefs about teaching demands, external resources, and potential barriers (Tschannen-Moran et al., 1998). Low teacher self-efficacy has been linked to job dissatisfaction, low teacher commitment, and intentions to quit (Brouwers & Tomic, 2000; Chan et al., 2008; Skaalvik & Skaalvik, 2011). Self-efficacy has also been found to positively predict perceived classroom management among early career teachers (Lazarides et al., 2020). Previous studies have found burnout and self-efficacy to be inversely related, with self-efficacy potentially serving as a resource and protective factor against burnout (Aloe et al., 2014; Dicke et al., 2014).

Burnout is defined as a prolonged response to chronic stressors over time (Maslach & Jackson, 1981; Maslach et al., 2001); in the education literature, it has been linked with job dissatisfaction and a desire to leave the workforce and/or change jobs (Pines & Keinan, 2005). Early career teachers are at particular risk for high burnout and leaving the teaching workforce, with teachers citing difficulties with classroom management and effectively supporting students who may engage in behaviors that interfere with learning as one of the most-common reasons for leaving the profession (Gavish & Friedman, 2010; Perrone et al., 2019; Skaalvik & Skaalvik, 2011). Based on the Job Demands-Resources Theoretical Model of Occupational Stress (Bakker & Demerouti, 2007; Dicke et al., 2014, 2018), when demands (e.g., managing student behavior) outweigh resources (e.g., classroom management skills) stress and burnout can occur.

### Impacts of the COVID-19 Pandemic

School closures resulting from the COVID-19 pandemic left schools, educators, students, and families in an unprecedented situation (Bradshaw et al., 2024a). A systematic review identified higher rates of burnout during the pandemic among K-12 teachers compared to before the COVID-19 pandemic (Westphal et al., 2022); higher burnout was associated with numerous COVID-specific factors, including learning loss among students and instructional difficulties with virtual/hybrid teaching (McDaniel et al., 2024; Sokal et al., 2020). Higher rates of burnout during COVID, as compared to pre-COVID, were also found to be associated with increased psychological distress and depressive symptoms arising from teaching anxiety, limited organizational supports, and dissatisfaction with decision making by administrators (Karakose et al., 2022; Pressley, 2021; Trinidad, 2021). Given the exacerbation of poor occupational health among teachers during COVID, difficulties with classroom management in the virtual teaching environment, and the increasing rates of teachers leaving the profession (Bacher-Hicks et al., 2023), it is necessary to investigate whether existing preventive interventions helped to promote positive occupational health outcomes during the pandemic.

### Overview of GBG + MTP

One preventive intervention primed to help early career teachers meet work-related demands is the PAX Good Behavior Game + MyTeachingPartner (GBG + MTP) intervention. GBG + MTP helps teachers develop skills and strategies to help meet challenging classroom demands, which in turn has translated into positive outcomes for both students (e.g., academic and behavioral; Allen et al., 2015; Ialongo et al., 2019; Johansson et al., 2020; Smith et al., 2021) and teachers (e.g., occupational health; Hopman et al., 2018). Specifically, GBG + MTP combines two evidence-based programs, GBG and MTP. GBG is a school-based intervention that leverages group behavioral contingencies to improve teachers’ classroom management skills and subsequently decrease student disruptive behavior (Barrish et al., 1969; Embry, 2002). GBG is based on social learning principles, with extensive research showing improvements in teacher, student, and teacher-student interaction outcomes (Hopman et al., 2018; Ialongo et al., 2019; Johansson et al., 2020; Smith et al., 2021). MTP is an evidence-based coaching intervention focused on improving the quality of student-teacher interactions (Allen et al., 2015). In the current study, MTP was largely used to help these early career teachers develop effective classroom practices and support the implementation of the GBG intervention, such that teachers received tailored, video-based feedback from trained coaches to further build their classroom management capacity. MTP was combined with GBG (i.e., GBG + MTP) to provide coaching support to the early career teachers as they worked to implement classroom management strategies (for additional information on the intervention and its implementation, see Braun et al., 2023; Tolan et al., 2020).

### GBG + MTP: The COVID Pandemic Follow-Up

Based on the promising initial findings (e.g., higher quality interactions with students, improved occupational health) from the original GBG + MTP trial (Downer et al., 2024; Tolan et al., 2020), additional funding was secured to follow these early career teachers to examine potential longer-term effects of the intervention. As such, a follow-up study was initiated for the 2019–2020 school year. However, just as this follow-up study was launched, there was an abrupt onset of the COVID pandemic, resulting in school closures and the pivot to online learning. Multiple modifications were made to the study design, including delaying data collection and removing in-person observations of teachers and students, due to limitations imposed by the school divisions and the universities related to in-person data collection. As a result, this follow-up was conducted over both the 2020–2021 and 2021–2022 school years, a time marked by increased turnover in the teaching profession, particularly over the course of the 2021–2022 school year (Bacher-Hicks et al., 2023).

These modifications to the study design, in addition to the COVID pandemic itself, could have introduced multiple forms of bias and created serious threats to inference regarding the efficacy of the GBG + MTP intervention if not properly addressed. From a measurement perspective, it is possible that the measurement properties of the outcomes changed when the mode of teaching was altered (i.e., in-person teaching vs. online) and different teaching demands during the pandemic as compared to prior years; this necessitates consideration of estimating scale scores that take into account differential item functioning for measurement occurring before vs. after the onset of COVID. It is also important to investigate and account for differential attrition from the study sample, given that teachers who remained in the sample during COVID may be systematically different from those who were lost to follow-up (Bacher-Hicks et al., 2023; Bell et al., 2013).

## Current Study

The GBG + MTP follow-up study conducted during the 2020–2021 and 2021–2022 school years provides a unique opportunity to explore the long-term effects of GBG + MTP on teacher occupational health, yet it was inherently complicated by the onset of the COVID pandemic. The overarching aim of this paper was to examine whether the GBG + MTP program longitudinally impacted levels of self-efficacy and burnout up to and during COVID. Consistent with the Job Demands-Resources Theoretical Model of Occupational Stress (Bakker & Demerouti, 2007; Dicke et al., 2014, 2018), the COVID pandemic likely exacerbated numerous existing precursors to teachers leaving the field; therefore, it is important to examine changes in self-efficacy and burnout during this vulnerable timeframe.

To address this primary aim, we employed a series of advanced methodological approaches to account for potential measurement noninvariance and missingness due to COVID. Specifically, we conducted moderated non-linear factor analyses (MNLFA; Bauer, 2017) to account for measurement noninvariance related to the onset of the pandemic among other factors. We also utilized a series of longitudinal analyses that varied in their assumptions regarding missing data mechanisms (i.e., missing-completely-at-random (MCAR); missing-at-random (MAR); not-missing-at-random (NMAR)) to assess the sensitivity of GBG + MTP effects in the presence of COVID-related dropout and retention. We discuss the strengths and limitations of the different missing data assumptions and approaches when analyzing RCT data impacted by the pandemic. As such, we consider the substantive implications of the RCT and contrast various approaches for managing missing data related to COVID, including measurement and missingness considerations, which has potentially important methodological insights for other research studies experiencing high rates of missingness because of COVID or other reasons.

## Methods

### Participants and Procedures

The original GBG + MTP trial launched in 2013 with three consecutive cohorts of early career teachers (*n* = 188, grades K-3) who were in their first three years of teaching (94% Female, 76% White, 58% first year teaching; Table 1). Teachers were randomly assigned to either the intervention condition (i.e., GBG + MTP; *n* = 94 teachers) or control condition (*n* = 94), and teacher- and student-outcomes were assessed pre-intervention (i.e., Cohort 1: Fall 2013, Cohort 2: Fall 2014, Cohort 3: Fall 2015), post-intervention (i.e., Cohort 1: Spring 2014, Cohort 2: Spring 2015, Cohort 3: Spring 2016), and 1-year post-intervention (i.e., Cohort 1: SY 2014-15, Cohort 2: SY 2015-16, Cohort 3: SY 2016-17; see supplementary materials) via observer-report, teacher-report, and standardized achievement scores. To be included in the original RCT study sample, teachers needed to complete pre-test data and persist through randomization. Additional information about the original GBG + MTP trial, including a more extensive, cohort-specific, CONSORT figure, can be found in Tolan et al. (2020).

**Table 1:** Description of teacher characteristics by intervention condition over time

	Baseline/Time 1			COVID Year 1/Time 5		COVID Year 2/Time 6	
	Total (*N* = 188)	Intervention (*n* = 94)	Control (*n* = 94)	Intervention (*n* = 36)	Control (*n* = 59)	Intervention (*n* = 25)	Control (*n* = 47)
	N (%)	N (%)	N (%)	N (%)	N (%)	N (%)	N (%)
Gender: Baseline*							
Female	176 (94%)	87 (93%)	89 (95%)	34 (94%)	55 (93%)	24 (96%)	44 (94%)
Male	12 (6%)	7 (7%)	5 (5%)	2 (6%)	4 (7%)	1 (4%)	3 (6%)
Race/Ethnicity: Baseline*							
African American/Black	42 (22%)	16 (17%)	26 (28%)	6 (17%)	18 (30%)	3 (12%)	15 (32%)
Hispanic/Latino/a/x	1 (0.5%)	0	1 (1%)	0	1 (2%)	0	1 (2%)
White	142 (75.5%)	75 (80%)	67 (71%)	29 (80%)	40 (68%)	21 (84%)	31 (66%)
Other/Multiple	3 (2%)	3 (3%)	0	1 (3%)	0	1 (4%)	0
Grade Level: Currently Teaching							
Pre-K	NA	NA	NA	1 (3%)	2 (3%)	1 (4%)	2 (4%)
Kindergarten	44 (23.4%)	21 (22%)	23 (24%)	6 (17%)	10 (17%)	6 (24%)	11 (23%)
1st grade	55 (29.3%)	28 (30%)	27 (29%)	4 (11%)	11 (19%)	3 (12%)	5 (11%)
2nd grade	42 (22.3%)	24 (26%)	18 (19%)	2 (6%)	7 (12%)	2 (8%)	6 (13%)
3rd grade	45 (23.9%)	20 (21%)	25 (27%)	7 (19%)	7 (12%)	3 (12%)	5 (11%)
4th−6th grade	NA	NA	NA	6 (17%)	11 (19%)	3 (12%)	6 (13%)
Multiple Elementary**	2 (1.1%)	1 (1%)	1 (1%)	7 (19%)	10 (17%)	6 (24%)	9 (19%)
Middle School Only	NA	NA	NA	0	0	0	1 (2%)
High School Only	NA	NA	NA	0	1 (2%)	0	1 (2%)
Multiple ES/MS/HS	NA	NA	NA	3 (8%)	0	1 (4%)	1 (2%)
Year of Teaching: Baseline							
First year	109 (58%)	56 (60%)	53 (56%)	23 (64%)	35 (59%)	14 (56%)	27 (58%)
Second year	42 (22.3%)	20 (21%)	22 (24%)	7 (19%)	14 (24%)	5 (20%)	10 (21%)
Third year	37 (19.7%)	18 (19%)	19 (20%)	6 (17%)	10 (17%)	6 (24%)	10 (21%)
	Mean (SD)	Mean (SD)	Mean (SD)	Mean (SD)	Mean (SD)	Mean (SD)	Mean (SD)
FARMS: Baseline*	79% (21%)	80% (0.21)	79% (0.22)	76% (0.21)	76% (0.25)	80% (0.15)	77% (0.26)
School Enrollment: Baseline*	515.22 (167.68)	522.87 (164.52)	507.56 (171.13)	535.72 (183.38)	532.56 (192.39)	552.20 (195.12)	507.91 (174.87)

*Indicates information was obtained at baseline and not available during COVID follow up waves. **In the baseline sample, multiple elementary refers to multiple grades taught within K-3. ES/MS/HS = Elementary School/Middle School/High School. FARMS = percentage of students receiving free and reduced meals. No significant differences (*p* < .05) between intervention and control groups at Time 1, Time 5, or Time 6

During the initial RCT and 1-year follow-up, participating teachers completed self-report measures online prior to randomization and attendance at the training (i.e., pre-test; Time 1), at the end of Spring of the first year of participation (i.e., post-intervention; Time 2), the subsequent Fall (i.e., Time 3), and during the Spring of the 1-year follow-up year (i.e., Time 4). During the Spring of 2021 (i.e., COVID Year 1; Time 5), teachers across all cohorts who had not actively withdrawn or become ineligible by Time 4 (i.e., Spring 1-year follow-up) were sent an email seeking their interest in participating in a follow-up survey via Qualtrics. In the Spring of 2022 (i.e., COVID Year 2; Time 6), teachers across all cohorts who were still teaching in 2021 and agreed to future contact were re-contacted to seek their participation in a similar follow-up survey. All teachers who provided their consent completed an initial screening in the online survey to indicate whether they were still actively in a teaching role. Participants who responded to the follow-up survey and were still in a teaching role (i.e., Time 5: *n* = 95; Time 6: *n* = 72) were asked a similar set of questions from the teacher survey included in the original trial, along with additional questions regarding sustained use of GBG + MTP and the perceived impacts of the pandemic on teaching practices, student outcomes, and personal experiences. Participants who responded to the survey but were no longer teaching (*n* = 20) were asked a series of questions about factors that contributed to their decision to leave the teaching profession but were not included in the follow-up study analyses on burnout and self-efficacy. All study phases were approved by the university’s Institutional Review Board. The study was pre-registered on the Registry of Efficacy and Effectiveness Studies (REES #21080), and the data was archived with the University of Virginia Dataverse (Bradshaw et al., 2024b).

### Measures

#### Burnout

 Four items from the Maslach Burnout Inventory (Maslach et al., 1997) were included in the burnout composite scale. Items were measured on a 4-point Likert scale, ranging from 1 to 4 (i.e., *Strongly Disagree* to *Strongly Agree*). The teachers reported on their experiences of the *emotional exhaustion* elements of burnout related to teaching (e.g., “I feel emotionally drained from my work”, “I feel used up at the end of the work day”). MNLFA scores were estimated to take into account differential item functioning (DIF) by wave. No statistically significant DIF by intervention condition was detected. The scale showed strong internal consistency across waves under Classical Test Theory (Cronbach’s α range = 0.82–0.94).

#### **Self-Efficacy**

 We assessed four items from Hoy and Woolfolk’s (1993) teaching self-efficacy measure. The items (e.g., “If I really try hard, I can get through to even the most difficult or unmotivated students” and “I can manage almost any student behavior problem”) were measured on a 4-point Likert scale (i.e., *Strongly Disagree* to *Strongly Agree*). The items were selected to be specific *to efficacy regarding the handling of student behavioral problems* rather than general teaching efficacy. No DIF was identified based on intervention condition or wave, so we proceeded with traditional factor scores in the analyses. The scale showed strong internal consistency across waves (Cronbach’s α range = 0.84–0.89).

#### Sociodemographic Information

Teachers responded to a series of survey questions about their demographic information (e.g., age range, race/ethnicity, gender identity, grade taught, years in the role). School-level demographic data was also obtained at baseline regarding the characteristics of the schools in which they taught during the initial intervention year (e.g., % of students receiving free and reduced meals (FARMS), total school enrollment).

## Missing Data

**Fig. 1:**
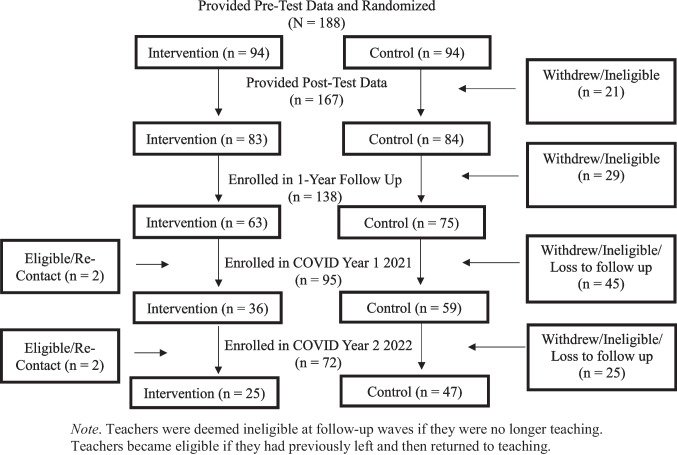
CONSORT

There was some attrition from the study between the first year and the follow-up year, such that 57% (*n* = 54) of intervention teachers and 77% (*n* = 72) of control teachers were followed from Time 1 through Time 4 (i.e., Pre-test through Spring 1-year follow-up; Fig. 1 CONSORT). During the first wave of COVID follow-up data collected in Spring of 2021 (i.e., Time 5), 38% (*n* = 36) of intervention teachers and 63% (*n* = 59) of control teachers responded to the survey and were still in a teaching role. During COVID Year 2 (i.e., Time 6), 27% (*n* = 25) of intervention teachers and 50% (*n* = 47) of control teachers responded to the survey and were still in a teaching role. Given these high rates of attrition, some of which may have been observed in later follow-ups because of the impacts of COVID, we investigated whether baseline teacher- and/or school-level factors were associated with study dropout. As a result of the high rates of missingness during the two COVID follow-up waves, we explored a variety of approaches to account for missing data under varying missing data assumptions.

### Statistical Analysis

#### Moderated Non-Linear Factor Analysis

 Given the many drawbacks of total scores (Lord et al., 1968; McNeish & Wolf, 2020; McNeish, 2023), we estimated scale scores accounting for differences in the relative weights of each item and measurement non-invariance/differential item functioning (DIF) due to COVID effects on assessment (among other factors). DIF can occur when individuals across time or from different groups who have the same level of the latent construct (e.g., burnout) have differing probabilities of endorsing an item. When not addressed, DIF can result in distorted scale scores, which can subsequently bias estimates of intervention effects (Brincks et al., 2018; Howe et al., 2019; Soland, 2022). To address these concerns, Moderated Non-Linear Factor Analysis (MNLFA) was employed to estimate scale scores accounting for DIF, which allows for both intercept and loading DIF to be modeled for continuous and categorical moderators. MNLFA scores improve upon traditional total scores by taking into account differences in both item intercepts (i.e., conditional prevalence of each item) and item factor loadings (i.e., the association between one item and the remaining pool of items). In addition, MNLFA allows for DIF to be modeled over time, which is particularly salient given the co-occurrence of the onset of COVID with changes in study design. We explored the potential impact of both intervention condition and wave on intercept and loading DIF for burnout and self-efficacy.

First, we confirmed the unidimensionality of both the burnout and self-efficacy subscales through separate 1-factor CFAs using weighted least squares estimation for categorical variables. We then tested the equal weighting assumption of total scores by comparing a factor model with constrained factor loadings to an unconstrained model. Once the best fitting model was identified, we conducted a series of MNLFA models in which DIF was examined separately for each subscale item. We examined whether intervention condition (i.e., GBG + MTP vs. control) or dummy coded wave variables, with baseline as reference, contributed to DIF above and beyond their influence on the underlying latent factor (e.g., burnout). Any DIF parameter that was significant at the *p* < .05 level in the individual item models was retained and included in the penultimate global MNLFA model. For any covariates that were significant predictors of factor loadings, the corresponding intercept parameter was also retained even if the intercept parameter was not significant given that we assume the covariate is associated with both the item loading and intercept (Gottfredson et al., 2019). The final global MNLFA model retained only those significant DIF parameters and corresponding intercept parameters that remained significant after combining all item parameters from individual item DIF analyses in the penultimate model (See supplementary material for DIF parameters). The final global MNLFA scale scores were exported from Mplus and used in all subsequent growth model and LCPMM analyses.

#### Additional Missing Data Assumptions with COVID-Impacted Missingness

The limitations of methods of modeling missing data under the MCAR assumption (e.g., complete case analysis) have long been known (see e.g., Rubin, 1976) and have largely been eschewed for methods with more flexible assumptions about the missing data mechanism: (a) that missingness is predictable by data that are observed (and the missing data “model” can be ignored) under the MAR assumption (e.g., full information maximum likelihood, multiple imputation) or (b) that a model for the probability of missingness has to be specified explicitly and jointly with the outcome model under NMAR (e.g., selection models, pattern mixture models; Enders, 2023). However, it is likely that the standard missing data assumptions may require additional considerations when thinking about the potential impacts of COVID on missingness.

First, there may be *two* types of missingness: (a) participants who were lost to follow-up *who would have been lost to follow-up regardless of COVID* (i.e., “always missing”) and (b) participants who were lost to follow-up *because of COVID* who would not have missed the assessment otherwise (i.e., “COVID missing”). Each of these types of participants may have observed variables from earlier assessments that would be useful in reducing the relation between their probability of missingness/loss to follow-up and their actual status on the outcome to near 0 (i.e., “functional” MAR; Collins et al., 2001). For example, teachers may have dropped out of the study, and missed follow-up assessment(s), *because of higher burnout*. If there are variables in the missing data model that are predictive of both dropout and burnout, then the missingness is MAR; if there remains a residual relation between dropout and the values of burnout that are missing, then data are NMAR. It is unclear as to whether “always missing” participants and “COVID missing” participants have different sets of variables that are predictive of missingness and/or are MAR versus NMAR. A second issue relates to differences among participants who *did* complete assessments during COVID, as these participants may be systematically different from other participants (particularly the COVID missing participants) on observed values on both covariates and outcomes; such participants presumably may be functioning better than other participants and/or came into the study at lower risk and be more likely to comply with study protocols. These participants might be termed “Present Not-at-Random”, much like “Always Takers” in non-compliance modeling (Frangakis & Rubin, 1999).

#### Proposed Missing Data Methodologies for the Present Study

Failing to address both general missingness and systematic missingness due to high burnout/self-efficacy itself and missingness due to COVID could bias intervention effect estimates (Lin et al., 2004; Roy, 2003). As a sensitivity analysis, and as a demonstration of addressing missingness potentially due to COVID-specific factors, we compared and contrasted multiple growth and mixture modeling approaches that varied in their assumptions regarding missing data mechanisms (i.e., MCAR, MAR, NMAR). First, a complete case analysis/intent-to-treat approach, which assumes MCAR, was used. Next, three different approaches were used that assume MAR: Complete Case Analysis with Inverse Probability of Participation Weighting (CCA/IPPW; Metten et al., 2022), Complete Case Analysis with Inverse Probability of Exposure Weighting (CCA/IPEW), and conventional Full Information Maximum Likelihood (FIML) under intent-to-treat. IPPW incorporates differential weighting for those who participated in the follow-up survey compared to those who did not, using covariates that help to predict an individual’s probability of being a “responder” (i.e., participating in the follow-up survey wave in consideration) while IPEW reweights the complete case sample to be balanced on covariates measured at baseline if there is differential dropout or loss to follow-up across intervention conditions.

For all MCAR and MAR approaches, we conducted several, separate growth models in which we examined teacher outcomes (i.e., burnout, self-efficacy) through COVID Year 1 (i.e., Times 1–5). We also attempted to explore impacts through COVID Year 2 (i.e., Times 1–6), although this was complicated in complete case models due to high levels of attrition and resulting sample size restrictions. We subsequently examined the impact of GBG + MTP on change in teacher outcomes over time (i.e., effect of GBG + MTP on the growth/slope of burnout/self-efficacy over time). We estimated a series of growth models (i.e., linear, quadratic, COVID-years freely estimated) to determine the best fitting model (RMSEA < 0.06, CFI/TLI > 0.95, Hu & Bentler, 1999). Freely estimating loadings during the COVID-years helps to account for both the time passage between the original trial and the COVID follow-up waves and potential non-linearity in trajectories over time. Across all missing data assumptions, the growth model with fixed loadings for Times 1–4 and freely estimated loadings at Time 5/COVID Year 1, and Time 6/COVID Year 2 when relevant, best fit the data. Effect sizes were calculated by multiplying the unstandardized beta coefficients for the linearly estimated waves (waves 1 to 4) by the duration (i.e., 3) plus the unstandardized beta coefficient(s) for the freely estimated waves, all divided by the baseline standard deviation of the measure (i.e., burnout/self-efficacy; Feingold, 2015). All MAR and MCAR models were adjusted for relevant covariates (e.g., cohort, grade taught, FARMS, total school enrollment).

**Fig. 2:**
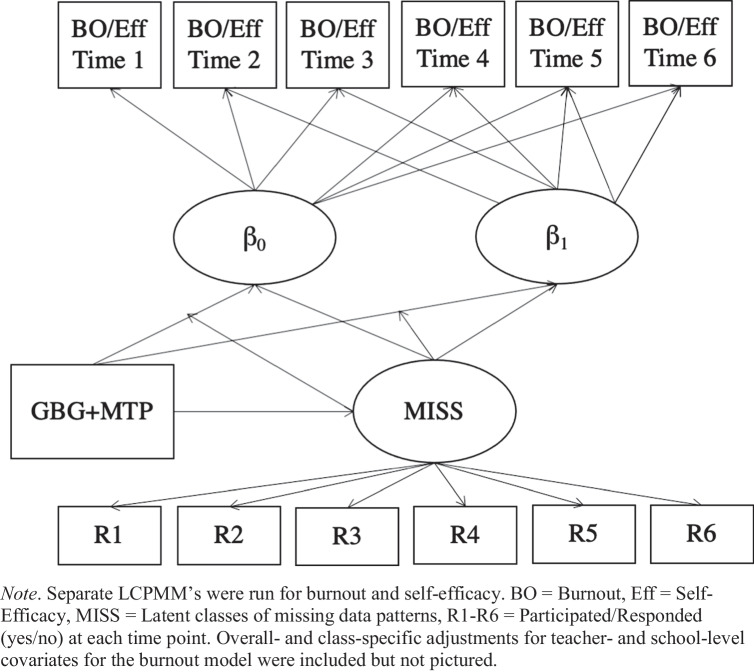
Latent Class Pattern Mixture Model Diagram

Lastly, we used latent class pattern mixture models (LCPMM) for modeling under the NMAR assumption (Lin et al., 2004; Morgan-López & Fals-Stewart, 2007; Roy, 2003; Fig. 2). LCPMMs are a latent class model, where each class is a joint function of both (a) differences in the probabilities of missingness across assessment missingness patterns and (b) differences in outcome trajectories. In LCPMMs, the model for missingness patterns is explicit, whereas there is no “model” for missingness (i.e., missingness is *ignorable*; Schafer & Graham, 2002) under the MCAR and MAR modeling frameworks. We conducted separate LCPMMs for burnout and self-efficacy through COVID Year 1 (i.e., Time 1–5) and COVID Year 2 (i.e., Time 1–6), and we examined the average intervention effect of GBG + MTP on burnout/self-efficacy across missingness classes. Given the potential impact of intervention condition on missingness class, we added an additional relationship of GBG + MTP predicting class membership.

## Results

### Differential Attrition

Although the intervention and control groups were balanced on demographic factors, burnout (*t*(186) = 0.376, *p* = .708, *d* = 0.06), and self-efficacy (*t*(78) = 1.867, *p* = .064, *d* = 0.28), at baseline (Table 1), exploratory analyses indicated that the intervention condition (i.e., intervention vs. control), working in a school with a higher percentage of students receiving free and reduced lunch (FARMS), and poor occupational health (e.g., low self-efficacy) were all significantly associated with teacher attrition from the study over time (Table 2). Neither years teaching nor years since receiving the intervention were associated with differential attrition. There was also a significant interaction between GBG + MTP and self-efficacy, adjusting for teacher- and school-level demographics. Intervention teachers with low self-efficacy at baseline were more likely to remain in the study compared to control teachers with similar levels of self-efficacy at baseline when examining teachers who remained at Time 5 (β = 0.004, *p* < .01) and Time 6 (β = 0.003, *p* = .03). This suggested that teachers working in lower resourced schools and with poorer occupational health were more likely to attrit from the sample, as well as a potentially buffering role of GBG + MTP on study dropout among a certain subset of teachers. Given these findings, not considering these significant differences could lead to biased conclusions given that teachers who remained in the sample during COVID were not representative of the original sample.

**Table 2:** Description of differential attrition of teachers over time

	Frequencies			Frequencies		
	Dropped Before COVID Year 1/Time 5 (*n* = 93)	Participated COVID Year 1/Time 5 (*n* = 95)	χ² (*p*-value)	Dropped Between Time 5 and Time 6 (*n* = 23)	Participated COVID Year 2/Time 6 (*n* = 72)	χ² (*p*-value)
Intervention Condition						
GBG + MTP	58 (62%)	36 (38%)	12.26 (< 0.001)	11 (48%)	25 (35%)	0.57 (0.45)
Control	35 (38%)	59 (62%)		12 (52%)	47 (65%)	
Gender*						
Female	87 (94%)	89 (94%)	0.00 (1.00)	21 (91%)	68 (94%)	0.29 (0.59)
Male	6 (6%)	6 (6%)		2 (9%)	4 (6%)	
Race/Ethnicity*						
African American/Black	18 (19%)	24 (25%)	2.44 (0.49)	6 (26%)	18 (25%)	0.85 (0.84)
Hispanic/Latino/a/x	0 (0%)	1 (1%)		0 (0%)	1 (1.4%)	
White	73 (78%)	69 (73%)		17 (74%)	52 (72.2%)	
Other/Multiple	2 (2%)	1 (1%)		0 (0%)	1 (1.4%)	
Grade Taught*						
Kindergarten	23 (25%)	21 (22%)	0.23 (0.99)	1 (4%)	20 (28%)	4.62 (0.33)
1st grade	28 (30%)	27 (28%)		7 (30%)	20 (28%)	
2nd grade	19 (20%)	23 (24%)		7 (30%)	16 (22%)	
3rd grade	22 (24%)	23 (24%)		8 (35%)	15 (21%)	
Multiple K-3	1 (1%)	1 (1%)		0 (0%)	1 (1%)	
Year of Teaching*						
First year	51 (55%)	58 (61%)	0.91 (0.64)	17 (74%)	41 (57%)	6.55 (0.16)
Second year	21 (23%)	21 (22%)		6 (26%)	15 (21%)	
Third year	21 (23%)	16 (17%)		0 (0%)	16 (22%)	
	Mean (SD)	Mean (SD)	*p*-value	Mean (SD)	Mean (SD)	*p*-value
FARMS*	83% (18%)	76% (24%)	0.03	73% (26%)	78% (22%)	0.32
School Enrollment*	499.23 (144.36)	531.20 (187.39)	0.19	523.29 (182.02)	566.26 (206.33)	0.34
Baseline Burnout	0.13 (0.86)	−0.09 (0.87)	0.10	0.04 (0.93)	−0.14 (0.84)	0.39
Baseline Self-Efficacy	−0.23 (0.95)	0.54 (0.84)	< 0.001	0.70 (0.99)	0.46 (0.46)	0.25

FARMS = percentage of students receiving free and reduced meals. *Indicates information obtained from baseline.

### Burnout

#### Preliminary Tests of Model Fit

We initially conducted a conventional test for unidimensionality across all waves of data for the items in the burnout subscale, which met some but not all standards for adequate fit (i.e., comparative fit index (CFI) = 0.99, root mean square error of approximation (RMSEA) = 0.17, standard root mean squared residual (SRMR) = 0.02; Millsap & Kwok, 2004). Given that sub-standard fit in the overall model may be an indicator of differential item functioning over time (Teresi & Fleishman, 2007), we proceeded to test whether a single factor adequately fit the items when conducted within wave-specific subsets of the data. Across all waves, we observed that a conventional single factor model fit the data (e.g., Time 1: CFI and TLI = 1.0, RMSEA < 0.01, SRMR = 0.01; Time 2: CFI = 1.0, TLI = 0.99, RMSEA = 0.03, SRMR = 0.01). We then compared the baseline/time 1 model to a model where factor loadings were constrained to be equal, which is the psychometric model that is assumed when using total scores (McNeish, 2023; McNeish & Wolf, 2020). This constrained model fit significantly worse compared to the conventional single factor model (χ² (3) = 25.56, *p* < .001). Given that the conventional model fit significantly better than the total score “analog” model and we observed differences in fit over time, we proceeded with conducting a moderated non-linear factor analysis to estimate DIF-adjusted factor scores, with an emphasis on DIF over time based on the pattern of fit statistics noted above.

#### MNLFA

 When exploring the dimensionality of the burnout latent factor, time-specific differences in item loadings and intercepts were observed. More specifically, during pre-COVID data collection (i.e., Time 1–4), two items of the burnout scale (i.e., item 1: “I feel burned out from my work”, item 3: “I feel emotionally drained from my work”) had higher factor loadings than the other two items (i.e., item 2: “I feel like I am at the end of my rope”, item 4: “I feel used up at the end of the work day”) that comprise the scale (e.g., > 0.90 vs. 0.50–0.70 respectively), whereas during COVID (i.e., Time 5–6), all items had high factor loadings (i.e., > 0.90). To further explore potential DIF across waves and intervention condition, MNLFA models were fit with time centered at baseline (See supplementary material for DIF parameters). In the final MNLFA model, Time 4–6 showed significant intercept DIF for item 1, while Time 5 and 6 also showed significant intercept DIF for item 2. Time 2 (i.e., post-intervention) showed significant loading DIF for item 4, while Time 3 and 4 showed significant loading DIF for item 2. The intervention was not a significant predictor of intercept or loading DIF across any item.

#### Complete Case Analysis

 In the growth model adjusted for covariates (i.e., teacher- and school-level demographic factors), there were no baseline differences in burnout between GBG + MTP and control teachers (β = 0.04, *p* = .870; Table 3) among teachers who remained in the sample from Time 1 through Time 5. In the same model, there was not a significant effect of GBG + MTP on change in burnout (i.e., growth parameter) from Time 1 through 5 (β = − 0.11, *p* = .269, effect size = − 0.35, 95% CI [−1.58, 0.89]). The complete case growth model for teacher burnout from Time 1 through Time 6 could not be estimated due to small sample size of teachers who participated across all 6 time points leading to poor fit and unreliable estimates.

**Table 3:** Growth models: GBG + MTP predicting burnout through COVID 2021 and COVID 2022

	Baseline to COVID Year 1 (Time 1 to Time 5)			Baseline to COVID Year 2 (Time 1 to Time 6)		
	Beta	SE	*p*-value	Beta	SE	*p*-value
Complete Case						
Int. (control)	−0.27	0.33	0.41	--	--	--
Slope (control)	0.14	0.13	0.28	--	--	--
GBG + MTP on Int.	0.04	0.22	0.87	--	--	--
GBG + MTP on Slope	−0.11	0.10	0.27	--	--	--
IPPW						
Int. (control)	−0.66	0.38	0.09	−0.88	0.59	0.14
Slope (control)	0.14	0.22	0.53	−0.39	0.22	0.07
GBG + MTP on Int.	0.03	0.65	0.97	−0.09	0.22	0.70
GBG + MTP on Slope	−0.11	0.33	0.75	−0.11	0.09	0.22
IPEW	--	--	--	--	--	--
FIML						
Int. (control)	−0.35	0.21	0.10	−0.28	0.21	0.18
Slope (control)	0.14	0.10	0.15	0.09	0.08	0.29
GBG + MTP on Int.	0.21	0.12	0.08	0.18	0.12	0.12
GBG + MTP on Slope	−0.13	0.06	**0.02**	−0.12	0.05	**0.03**
LCPMM						
Avg. GBG + MTP on Slope: 2 Class Model	−0.10	0.07	0.20	−0.09	0.09	0.23

IPPW = Inverse Probability of Participation Weights, IPEW = Inverse Probability of Exposure Weights, LCPMM = Latent Class Pattern Mixture Model. Avg. = Average effect of GBG + MTP on slope across classes. Bold text indicates *p* < .05. All models adjusted for relevant teacher- and school-level demographic factors

#### IPPW and IPEW

 After incorporating inverse probability weights for participation at Time 5 into the adjusted model with complete cases, there was not a significant impact of GBG + MTP on the growth parameter of burnout from Time 1 through Time 5 (β = − 0.11, *p* = .754, effect size = − 0.25, 95% CI [−1.55, 1.05]). There was similarly no impact of GBG + MTP on growth of burnout from Time 1 through Time 6 after incorporating IPP weights for participation at Time 6 into the model (β = − 0.11, *p* = .216, effect size = 0.29, 95% CI [−1.51, 2.10]). There were also no baseline differences between GBG + MTP and control teachers’ burnout at baseline in the IPPW models. The IPEW approach was not feasible due to lack of school-specific covariates available during the COVID follow-up waves (e.g., FARMS, total student enrollment, in-person vs. virtual instruction), which precluded our ability to recreate balance on important covariates between the intervention and control groups.

#### FIML

When incorporating all datapoints across all teachers in the growth model and utilizing FIML for missing data, there was a significant effect of GBG + MTP on burnout (i.e., growth parameter) from Time 1 through Time 5 (β = − 0.13, *p* = .020, effect size = − 0.07, 95% CI [−1.00, 0.84]). There were no baseline differences between the GBG + MTP and control teachers’ levels of burnout in this model (β = 0.21, *p* = .084). These findings indicated a non-significant positive slope (i.e., increasing burnout over time) for control teachers, whereas GBG + MTP teachers indicated a reduction in burnout over time. Similar findings were observed for teacher burnout from Time 1 through Time 6, such that we observed a significant impact of GBG + MTP on change in burnout from Time 1 to Time 6 (β = − 0.12, *p* = .03, effect size = 0.25, 95% CI [−1.22, 1.69]). Teachers in the GBG + MTP condition demonstrated decreasing burnout from Time 1 through Time 6, while control teachers showed non-significant worsening burnout across time.

#### LCPMM

Based on fit statistics and conceptual utility (i.e., decreasing BIC, significant LMR *p*-value; See supplementary material), we interpret model parameters for the 2-class model for Baseline through COVID Year 1 (i.e., Time 1 to 5) and Baseline through COVID Year 2 (i.e., Time 1 to 6). In the COVID Year 1 model, class 1 teachers were likely to participate through COVID Year 1, whereas class 2 was comprised of teachers who dropped out between Time 2 and 3 (i.e., original trial and 1-year follow-up). Intervention condition was significantly associated with missingness class (β = −0.71, *p* = .042), such that intervention teachers were more likely to be in class 2. Neither the average intervention effect of burnout across classes (β = − 0.10, *p* = .198) nor within classes (class 1: β = −0.05, *p* = .350; class 2: β = 0.24, *p* = .206) was significant. In the COVID Year 2 model, class 1 teachers were likely to remain in the sample through Time 6, whereas teachers in class 2 were most likely to drop out of the study between Time 2 and 3 (i.e., original trial and 1-year follow-up). Teachers in the GBG + MTP condition were more likely to be in class 2 than control teachers (β = −0.88, *p* = .014). Neither the average intervention effect of burnout across classes (β = − 0.09, *p* = .233) nor within classes (class 1: β = −0.06, *p* = .188; class 2: β = − 0.23, *p* = .505) was significant.

### Self-Efficacy

#### Preliminary Tests of Model Fit

We identified that a conventional, single factor model fit the self-efficacy items well both across and within waves (e.g., across time: CFI = 1.0, RMSEA < 0.01, SRMR = 0.01; time 1: CFI = 1.0, RMSEA < 0.01, SRMR = 0.01), and this model fit significantly better than the total score “analog” model (e.g., RMSEA = 0.15; χ² (3) = 20.79, *p* < .001). Given that the conventional model fit the data better than the constrained model and initial exploration demonstrated no evidence of DIF, we proceeded with unadjusted factor scores. All subsequent MAR and MCAR models were adjusted for relevant teacher- and school-level demographic factors.

#### Complete Case Analysis

Among all teachers who participated at Time 1 through 5, there were no baseline differences between GBG + MTP and control teachers’ level of self-efficacy (β = 0.14, *p* = .506; Table 4), nor was there an impact of GBG + MTP on change in self-efficacy from Time 1 through Time 5 (β = 0.03, *p* = .321, effect size = 0.59, 95% CI [−0.66, 1.83]). Similar to burnout, the complete case growth model for self-efficacy from Time 1 through Time 6 could not be estimated due to small sample size of teachers who participated across all 6 time points.

**Table 4:** Growth models: GBG + MTP predicting self-efficacy through COVID 2021 and COVID 2022

	Baseline to COVID Year 1 (Time 1 to Time 5)			Baseline to COVID Year 2 (Time 1 to Time 6)		
	Beta	SE	*p*-value	Beta	SE	*p*-value
Complete Case						
Int. (control)	−0.57	0.25	**0.03**	--	--	--
Slope (control)	0.08	0.04	0.07	--	--	--
GBG + MTP on Int.	0.14	0.21	0.51	--	--	--
GBG + MTP on Slope	0.03	0.03	0.32	--	--	--
IPPW						
Int. (control)	−0.66	0.23	**0.004**	−0.65	0.24	**0.01**
Slope (control)	0.09	0.08	0.29	0.19	0.07	**0.01**
GBG + MTP on Int.	0.18	0.25	0.46	−0.26	0.27	0.34
GBG + MTP on Slope	0.02	0.04	0.55	0.14	0.08	0.09
IPEW	N/A	N/A	N/A	N/A	N/A	N/A
FIML						
Int. (control)	−0.38	0.15	**0.01**	− 0.39	0.16	**0.01**
Slope (control)	0.06	0.03	**0.02**	0.06	0.03	**0.04**
GBG + MTP on Int.	−0.10	0.11	0.37	− 0.09	0.11	0.41
GBG + MTP on Slope	0.02	0.02	0.23	0.10	0.01	0.40
LCPMM						
Avg. GBG + MTP on Slope: 2 Class Model	0.001	0.01	0.88	0.01	0.03	0.85

IPPW = Inverse Probability of Participation Weights, IPEW = Inverse Probability of Exposure Weights, LCPMM = Latent Class Pattern Mixture Model. Avg. = Average effect of GBG + MTP on slope across classes. Bold text indicates *p* < .05. All models adjusted for relevant teacher- and school-level demographic factors

#### IPPW and IPEW

After incorporating IPP weights for participating up to Time 5 into the complete case growth model, there was no effect of GBG + MTP on self-efficacy from Time 1 through Time 5 (β = 0.02, *p* = .552, effect size = 0.58, 95% CI [−0.93, 2.09]), nor were there baseline differences in self-efficacy between GBG + MTP and control teachers (β = 0.18, *p* = .455). There was similarly no impact of GBG + MTP on self-efficacy following teachers from Time 1 through Time 6 (β = 0.14, *p* = .089, effect size = 0.18, 95% CI [−2.59, 2.93]), nor were there baseline differences between GBG + MTP and control teachers (β = −0.26, *p* = .339). The IPEW model was not able to be estimated as explained previously, given the limited available school-level characteristics at follow-up waves.

#### FIML

When employing FIML to account for partially missing data when examining change in self-efficacy from Time 1 through Time 5, there were no baseline differences in self-efficacy for GBG + MTP teachers compared to control teachers (β = −0.10, *p* = .367). In the same model, no effect of GBG + MTP on change in self-efficacy from Time 1 through Time 5 was observed (β = 0.02, *p* = .228, effect size = 0.33, 95% CI [−0.56, 1.23]). There was similarly no impact of GBG + MTP on change in self-efficacy from Time 1 through Time 6 (β = 0.10, *p* = .402 effect size = 0.31, 95% CI [−1.89, 1.80]). Across both models, there was a positive significant slope for all teachers’ improving self-efficacy over time (Time 1 to 5: β = 0.06, *p* = .022; Time 1 to 6: β = 0.06, *p* = .035).

#### LCPMM

 Moving from a 1- to 3-class model examining self-efficacy, we identified that, based on fit statistics, the 2-class models best fit the data for both Baseline through COVID Year 1 (i.e., Time 1 to 5) and Baseline through COVID Year 2 (i.e., Time 1 to 6). For the COVID Year 1 model, teachers in class 1 were likely to remain through COVID Year 1, while teachers in class 2 tended to drop out of the study between Time 2 and 3 (i.e., original trial and 1-year follow-up). There was no average effect of GBG + MTP on self-efficacy (β = 0.001, *p* = .882), nor were there class-specific GBG + MTP effects (class 1: β = 0.01, *p* = .592; class 2: β = −0.02, *p* = .475). Teachers in the GBG + MTP condition were more likely to be in class 2 compared to control teachers (β = −1.19, *p* = .011). In the COVID Year 2 model, class 1 teachers were likely to participate through both years of COVID follow-up data collection. Class 2 teachers were most likely to participate in the original trial year, somewhat likely to participate in the follow-up, and not likely to participate during either COVID year. There was no average GBG + MTP effects on self-efficacy (β = 0.01, *p* = .845), nor were there class-specific GBG + MTP effects (class 1: β = −0.001, *p* = .795; class 2: β = 0.02, *p* = .827) on self-efficacy. Intervention teachers were significantly more likely to be in class 2 compared to control teachers (β = −1.11, *p* < .001).

## Discussion

This study sought to compare and contrast different methodological approaches to examine the impact of the GBG + MTP intervention on teacher burnout and self-efficacy while accounting for missing data, study attrition among early career teachers, and other challenges resulting from the COVID pandemic. Prior research identified a clear increase in educator burnout during COVID (Westphal et al., 2022), yet there has been limited exploration of the impact of classroom management interventions on burnout and self-efficacy during the pandemic. Across the multiple modeling approaches under varying missing data assumptions, we identified mixed findings (i.e., both null and beneficial) for the impact of GBG + MTP on burnout overtime, although we did not observe an effect of GBG + MTP on self-efficacy. We discuss the implications of the intervention findings, as well as the benefits and limitations of these varying modeling approaches when analyzing RCT outcomes impacted by COVID. When interpreting analyses conducted among teachers who persisted up to and through the first year of COVID, special attention needs to be paid to whom these findings can be generalized.

### Differential Attrition

We identified multiple factors associated with differential attrition from the study among early career teachers participating in GBG + MTP, including teacher- (e.g., self-efficacy) and school-level (e.g., % of students receiving FARMS) factors, which may serve as important indicators of teachers at greatest risk for leaving the teaching workforce. Ignoring the presence of differential attrition by only using complete cases could lead to biased results, as teachers who remained in the sample worked in higher resourced schools and exhibited better occupational health at baseline. It is also likely that numerous COVID-specific factors not captured at baseline contributed to the large proportion of teachers that attrited from the sample between Time 4 and Time 5 (i.e., 1-year follow up and COVID Year 1). The subset of teachers who responded to the follow-up survey during the pandemic who had left teaching (*n* = 20) provided additional insight into some of the potential COVID-specific predictors of leaving the workforce. These former teachers indicated that serious disruptions to teaching due to COVID and difficulty coping with COVID-19 related challenges influenced their decision to leave teaching. They also noted other more commonly reported predictors of leaving the teaching workforce, including a lack of support from administrators, stress of managing student behavior, a lack of respect from students and parents, a lack of opportunity for career advancement, and low compensation. As a result, when analyzing and interpreting RCTs impacted by COVID, both baseline predictors of study attrition as well as novel, COVID-specific predictors should be considered as those who remained in the study are inherently different from those who dropped out.

### Burnout

Consistent with prior literature, teachers who participated in the follow-up survey reported worsening burnout during COVID (Westphal et al., 2022), independent of changes in the measurement properties of the burnout measure during COVID. Although we did not identify an intervention impact in the complete case and IPPW models, we did identify a beneficial intervention effect in the growth model employing FIML for partially missing data. Given the consistency of the growth parameter estimates across all models for the impact of GBG + MTP on burnout (i.e., β range = −0.11 to −0.13), it is likely that the complete case models were underpowered to detect a significant effect. This may be similarly true for the LCPMMs, as identifying the correct number of classes in mixture modeling is sensitive to sample size (Nylund et al., 2007). Additionally, findings from the 1-year follow-up study only showed a trend level impact of GBG + MTP on teacher distress (i.e., burnout and stress), while the most robust findings were among teachers working in higher risk conditions (e.g., classrooms with higher rates of student disruptive behavior; Downer et al., 2024). It may similarly be the case that GBG + MTP differentially impacts teachers working in higher risk classrooms, yet sample size restrictions precluded our ability to test similar moderating effects.

From a methodological perspective, these varied findings highlight the pressing need to consider multiple approaches to account for study design and missing data challenges when analyzing the effects of an RCT impacted by COVID (See Table 5). More specifically, the growth model was sensitive to how the missing data assumptions were handled, while the LCPMM shed light on differences in the impact of GBG + MTP on burnout depending on when teachers dropped out of the study. We also identified a potential shift in the underlying construct of burnout itself, evidenced by the changing intercepts and factors loadings comparing the items pre-COVID to during-COVID, which may have gone undetected had traditional total scores been utilized. From a substantive perspective, it is possible that GBG + MTP may have been particularly impactful for those teachers who had other characteristics that increased their likelihood of remaining in the study (i.e., moderated the relationship between teacher- or school-characteristics and burnout). It may also be the case that GBG + MTP led to reduced burnout, which subsequently increased the chances of remaining in the sample and potentially in the teaching workforce. The beneficial effects identified in the growth model with FIML demonstrate the potential for GBG + MTP to provide teachers with classroom management skills that have positive, long-term impacts on burnout. If not for the pandemic, we may have observed a stronger effect for intervention teachers, since GBG + MTP was designed to equip teachers with strategies to help manage their classroom in-person. However, teaching was primarily virtual or hybrid during the COVID follow-up years. Thus, the skills developed through GBG + MTP may have only partially helped teachers meet the unique demands of an online learning environment and the novel challenges associated with teaching through the pandemic.

**Table 5:** Comparison of Missing Data approaches

Approach	Missing Assumption	Strengths	Limitations	Interpretation
Growth Model with Complete Cases	MCAR	Most parsimonious approach if MCAR assumption holds	Biased estimates if differential attrition, sample size challenges	Effect of intervention on outcome over time among teachers who persisted assuming no differential attrition
Growth Model with IPP Weights	MAR	Responders “carry the weight” of the non-responders, weight calculation includes all participants	Outcome prediction model only includes complete cases	Effect of intervention on outcome over time among teachers who persisted, weighted by baseline covariates of both responders and non-responders
Growth Model with IPE Weights	MAR	Can “re-balance” the follow up sample on important covariates, accounts for changes in covariates (e.g., school-level factors)	Only possible if updated covariates available, still relies on complete cases	Difference in outcome by intervention condition, adjusting for covariates through re-weighting of conditions by time-varying covariates
Growth Model with FIML	MAR	Includes the whole sample over time, relies on existing data to estimate missing values for partially missing data	No separate missingness model, FIML may produce biased estimates	Effect of intervention on outcome across time accounting for partially missing data
Latent Class Pattern Mixture Model	NMAR	Includes the whole sample, includes separate missingness classes, estimates not distorted if data are actually MAR, provides average and class-specific treatment effects	Difficulties incorporating covariate interactions	Average weighted intervention effect on outcome accounting for differing patterns of missingness and partially missing data

MCAR = Missing Completely at Random, MAR = Missing at Random, NMAR = Not Missing at Random, IPP = Inverse Probability of Participation, IPE = Inverse Probability of Exposure, FIML = Full Information Maximum Likelihood

### Self-Efficacy

Consistent with prior research, we observed a slight increase in self-efficacy over time for teachers who remained in the study (Holzberger et al., 2013; Skaalvik & Skaalvik, 2017), although this did not vary between intervention and control teachers. These null intervention effects may be due to the changing needs for teachers to feel efficacious during COVID, especially as it pertains to classroom management. Previous literature has identified that difficulties with virtual instruction and managing student behavior in the virtual environment are predictors of poor occupational health during COVID (McDaniel et al., 2024; Sokal et al., 2020). The lack of intervention effect may also be due to the lack of available information regarding school-level characteristics during follow-up, which may have significantly changed from baseline through COVID due to the high rates of school-switching (i.e., over half of teachers changed schools throughout the original and follow-up study). The inability to adequately adjust for these school-level covariates during the COVID follow-up waves may have limited our ability to detect intervention effects. There may also have been residual effects of missing data that were not fully accounted for due to limitations in measured variables. For example, it may have been the case that teachers in the control condition dropped out of the study due to low self-efficacy, whereas intervention teachers dropped out due to unmeasured, COVID-specific factors (e.g., health concerns). These null findings have important implications for both intervention design (i.e., applicability of GBG + MTP to online teaching) and study design (i.e., need for continued collection of time-varying variables for early career teachers).

### Limitations and Future Directions

Beyond the study design limitations, there are lingering challenges when conducting follow-up studies during COVID, including unmeasured COVID-specific factors (e.g., lay-offs, contracting COVID, other health impacts of the pandemic, competing family priorities) that may lead to withdrawal from study participation. We used abbreviated measures of two widely used measures of efficacy and burnout to reduce burden on the teachers; these same abbreviated measures have been previously used in several prior studies (e.g., Pas et al., 2012). Future studies would benefit from collecting information on teachers’ intentions to both remain in the study sample as well as in the field of teaching to provide greater insight into predictors of both study and teaching workforce retention. In addition, the follow-up study delayed data collection due to the onset of the pandemic, which likely led to further loss of study participants due to the increasing time frame between the initial intervention and follow-up. Based on these findings and recent research, it is evident that COVID has lingering impacts on retention, social and emotional well-being, and academic achievement. Overall, detecting conditional effects in interventions affected by COVID may be particularly difficult due to reduction in sample sizes and diminished power. Additionally, each score generation (e.g., MNLFA) and modeling approach has methodological limitations (Table 5), and future studies should consider comparing findings across approaches to improve confidence in the findings. Continued research is needed to identify potential interventions that had or can have, with relevant tailoring to the peri-COVID learning environment, significant impacts on teacher burnout and self-efficacy. One “positive” consequence that may come from these challenges is the emphasis on transparency when discussing study samples and the handling of missing data and measurement.

## Conclusion

COVID-19 impacted both classroom instruction and analysis of school-based RCTs. The current study aimed to compare and contrast different methodological approaches under varying missing data assumptions to examine the long-term effects of GBG + MTP on teacher-reported burnout and self-efficacy. This study highlights the importance of considering the interpretation and external validity of RCT findings when the sample was impacted by differential attrition and missingness due to the COVID-19 pandemic. There will continue to be long-term consequences of the pandemic on teacher- and student-outcomes, and there will be a pressing need to continue focusing on transparency of reporting sample characteristics, design impacts, and handling of missing data.

## Supplementary Information

Below is the link to the electronic supplementary material.

ESM 1 (174 KB) 
