# Supplementary material for: Addressing Methodological Challenges in Follow-Up RCTs During the COVID-19 Pandemic: The Impact of the Good Behavior Game and MyTeachingPartner™ on Teacher Burnout and Self-Efficacy
Source: Prev Sci. 2025 Jan 2;27(1):79–95. doi: 10.1007/s11121-024-01757-9 (PMC12906517; doi:10.1007/s11121-024-01757-9)
Supplement: Supplementary file 1 — (174 KB) [file 11121_2024_1757_MOESM1_ESM.docx]

**Supplementary Tables and Figures**

**Table S1**

*MNLFA Parameters for Burnout*

| Item | Factor Loading | Threshold (τ; 0 to 1) | Threshold  (1 to 2) | Threshold  (2 to 3) | Tx λ  DIF | Time 1 λ  DIF | Time 2 λ  DIF | Time 3 λ  DIF | Time 4 λ  DIF | Time 5 λ  DIF | Time 6 λ  DIF | Tx τ  DIF | Time 1 τ  DIF | Time 2 τ  DIF | Time 3 τ  DIF | Time 4 τ  DIF | Time 5 τ  DIF | Time 6 τ  DIF |
| --- | --- | --- | --- | --- | --- | --- | --- | --- | --- | --- | --- | --- | --- | --- | --- | --- | --- | --- |
| 1. Burned out | 5.08 | -7.37 | -0.88 | 5.08 |  |  |  |  |  |  |  |  |  |  |  | 0.86 | 2.21 | 2.62 |
| 2. End of Rope | 2.46 | -1.22 | 2.78 | 5.67 |  |  |  | -.09 | .09 |  |  |  |  |  | 0.67 | 0.64 | 2.51 | 2.61 |
| 3. Emotionally Drained | 4.32 | -7.23 | -2.03 | 3.06 |  |  |  |  |  |  |  |  |  | .07 |  |  |  |  |
| 4. Used Up | 2.39 | -4.84 | -2.13 | 1.10 |  |  | -0.40 |  |  |  |  |  |  |  |  |  |  |  |

*Note*. Tx = Treatment condition (i.e., GBG+MTP or control), DIF = Differential Item Functioning.

**Table S2**

*LCPMM Fit Statistics for Burnout*

| Baseline to COVID Year 1 | | |  |  |  |  |
| --- | --- | --- | --- | --- | --- | --- |
|  | BIC | SSA-BIC | Adj. LMR *p*-value | Class 1 Size | Class 2 Size | Cass 3 size |
| 1 Class | 2653.99 | 2571.64 | -- | 184 (100%) |  |  |
| 2 Class | **2519.48** | **2367.46** | **<.001** | **123 (67%)** | **61 (33%)** |  |
| 3 Class | 2583.88 | 2362.18 | .27 | 34 (18%) | 60 (33%) | 90 (49%) |
| Baseline to COVID Year 2 | |  |  |  |  |  |
|  | BIC | SSA-BIC | Adj. LMR *p*-value | Class 1 Size | Class 2 Size | Cass 3 size |
| 1 Class | 3083.47 | 2994.79 | -- | 184 (100%) |  |  |
| 2 Class | **2909.06** | **2747.53** | **<.001** | **131 (71%)** | **53 (29%)** |  |
| 3 Class | 2935.65 | 2701.28 | <.001 | 55 (30%) | 51 (28%) | 78 (42%) |

*Note.* BIC = Bayesian Information Criteria, SSA-BIC = Sample Size Adjusted Bayesian Information Criteria, Adj. LMR = Adjusted Lo-Mendell Rubin Likelihood Ratio Test.

**Table S3**

*LCPMM Fit Statistics for Self-Efficacy*

| Baseline to COVID Year 1 | |  |  |  |  |  |
| --- | --- | --- | --- | --- | --- | --- |
|  | BIC | SSA-BIC | Adj. LMR *p*-value | Class 1 Size | Class 2 Size | Cass 3 size |
| 1 Class | 2530.71 | 2454.70 | -- | 184 (100%) |  |  |
| 2 Class | **2447.08** | **2307.72** | **<.001** | **118 (64%)** | **66 (36%)** |  |
| 3 Class | 2934.65 | 2738.28 | <.001 | 96 (52%) | 45 (25%) | 43 (23%) |
| Baseline to COVID Year 2 | | |  |  |  |  |
|  | BIC | SSA-BIC | Adj. LMR *p*-value | Class 1 Size | Class 2 Size | Cass 3 size |
| 1 Class | 2960.50 | 2878.15 | -- | 184 (100%) |  |  |
| 2 Class | **2830.85** | **2681.97** | **<.001** | **95 (52%)** | **89 (48%)** |  |
| 3 Class | 3300.07 | 3091.03 | <.001 | 75 (41%) | 55 (30%) | 54 (29%) |

*Note.* BIC = Bayesian Information Criteria, SSA-BIC = Sample Size Adjusted Bayesian Information Criteria, Adj. LMR = Adjusted Lo-Mendell Rubin Likelihood Ratio Test.

**Table S4**

*Probability of Participating at Each Timepoint for the 2-Class Models: Burnout and Self-Efficacy*

|  | Burnout | | | | Self-Efficacy | | | |
| --- | --- | --- | --- | --- | --- | --- | --- | --- |
|  | Baseline to CY1 | | Baseline to CY2 | | Baseline to CY1 | | Baseline to CY2 | |
|  | Class 1: “Low Attrition” | Class 2: “High Attrition” | Class 1: “Low Attrition” | Class 2: “High Attrition” | Class 1: “Low Attrition” | Class 2: “High Attrition” | Class 1: “Low Attrition” | Class 2: “High Attrition” |
| Time 1 | 98% | 100% | 98% | 100% | 98% | 99% | 97% | 99% |
| Time 2 | 98% | 60% | 97% | 57% | 100% | 59% | 96% | 74% |
| Time 3 | 99% | 3% | 92% | 5% | 95% | 13% | 89% | 41% |
| Time 4 | 91% | 6% | 88% | 0% | 83% | 12% | 76% | 38% |
| Time 5 | 71% | 12% | 72% | 0% | 71% | 15% | 98% | 0% |
| Time 6 | -- | -- | 55% | 0% | -- | -- | 75% | 0% |

*Note.* CY1 = COVID Year 1/Time 5, CY2 = COVID Year 2/Time 6.

**Figure S1.** Original Trial and Follow-Up Study Timeline


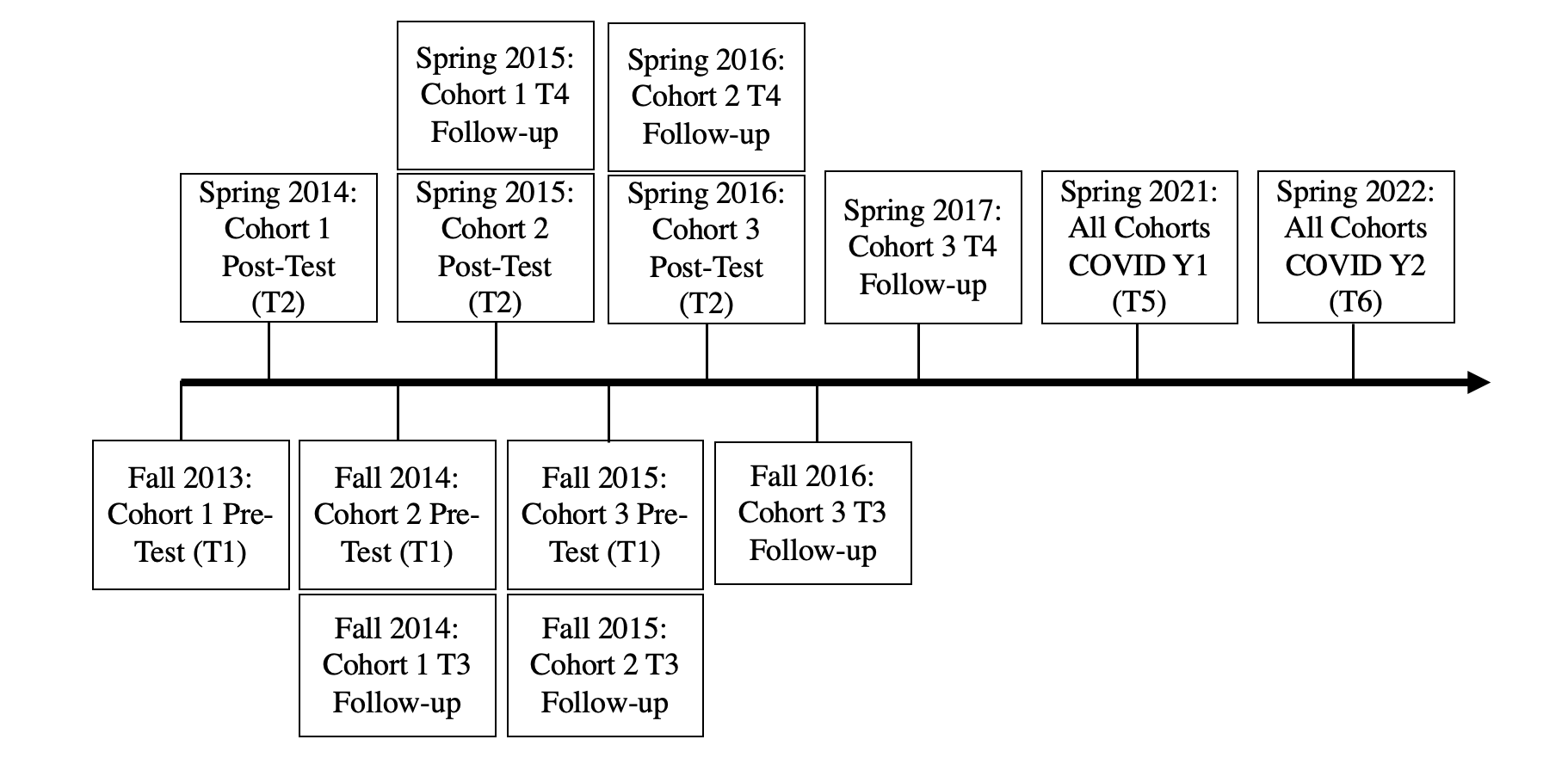


*Note*. T = Time, Y = Year.
